# Unilateral Hearing Loss and Auditory Asymmetry in Mitochondrial Disease: A Scoping Review

**DOI:** 10.3390/jcm13175044

**Published:** 2024-08-26

**Authors:** Marianna Manuelli, Andrea Migliorelli, Chiara Bianchini, Francesco Stomeo, Stefano Pelucchi, Elisabetta Genovese, Daniele Monzani, Silvia Palma, Andrea Ciorba

**Affiliations:** 1 ENT & Audiology Unit, Department of Neurosciences, University Hospital of Ferrara, 44124 Ferrara, Italy; 2 ENT and Audiology Department, University Hospital of Modena, 44121 Modena, Italy; 3 ENT and Audiology Department, University Hospital of Verona, 37126 Verona, Italy; 4 Audiology, Primary Care Department, Azienda Unità Sanitaria Locale (AUSL), 44121 Modena, Italy

**Keywords:** mitochondrial disease, hearing loss, deafness, unilateral hearing loss

## Abstract

**Background/Objectives**: Mitochondrial transfer RNA mutations are one of the most important causes of hereditary hearing loss in humans. In most cases, its presentation is bilateral and symmetrical; however, there are numerous cases of single-sided presentation or asymmetrical onset described in the literature that may represent a diagnostic challenge. The aim of this review is to present the evidence of auditory asymmetry in mitochondrial diseases, highlighting the possible presence of cases with atypical presentation. **Methods**: A review of the English literature to date on hearing loss and mitochondrial diseases was performed using PubMed, Scopus, and Google Scholar databases. The literature review was performed using the Preferred Reporting Items for Systematic Reviews and Meta-analysis (PRISMA) guidelines for scoping review. **Results**: A total of 10 full-text articles were included in this review, comprising 25 patients with single-sided or asymmetrical hearing loss associated with mitochondrial disease. **Conclusions**: Sensorineural hearing loss due to mitochondrial disease can represent a complex diagnostic challenge in cases of asymmetric or unilateral presentation. It is critical to recognize this clinical variant and to diagnose it in daily clinical practice.

## 1. Introduction

Hearing loss is the most common sensory organ deficit and affects about 1–2 per 1000 births and 13% of adult population worldwide [1,2].

Over half of cases of congenital hearing loss are genetically transmitted as autosomal recessive or dominant or through rarer mechanisms such as X-linked inheritance and mitochondrial DNA [1].

Hearing loss associated with mitochondrial disease is typically progressive, sensorineural, and affects all frequencies in a predominantly symmetrical manner, but there are cases of asymmetry that need to be known and recognized to allow early and timely diagnosis [2].

Mitochondria are organelles located in eukaryotic cell that are responsible for energy production [3]. In addition to their important function, they have the peculiarity of possessing their own genetic material named mitochondrial DNA (mtDNA). Since mitochondria are responsible for energy production, the first targets of their disruption are high energy-consuming organs and systems, such as the nervous system, retina, cochlea, muscle, kidney, and liver [3,4].

Mitochondrial diseases have a heterogeneous presentation and a progressive course, and hearing loss is a typical symptom [4].

Mitochondrial disorders can present as syndromic forms, such as Kearns-Sayre syndrome (KSS), MELAS (mitochondrial encephalomyopathy, lactic acidosis, stroke-like episodes) syndrome and MERRF (myoclonic epilepsy associated with ragged red fibers) syndrome, or as nonsyndromic forms, with hearing loss alone or paired with only one additional symptom [2,5].

The presentation of hearing loss in mitochondrial diseases is bilateral, symmetrical, and progressive in most cases, but there are a few known cases of unilateral or asymmetrical presentation that represent a major diagnostic challenge [2,5].

The purpose of this review is to highlight the non-negligible occurrence of asymmetric hearing loss or single-sided deafness in mitochondrial diseases reported in the literature and to increase awareness and clinical vigilance of these entities, as these presentations could lead to significantly delayed diagnosis.

To the best of our knowledge and because of the limited literature available, this is the first review study investigating asymmetric hearing loss in syndromic and nonsyndromic mitochondrial diseases and its clinical and genetic features.

## 2. Materials and Methods

A review of the English literature to date on mitochondrial diseases and hearing loss was performed using PubMed, Scopus, and Google Scholar databases.

**Figure 1:**
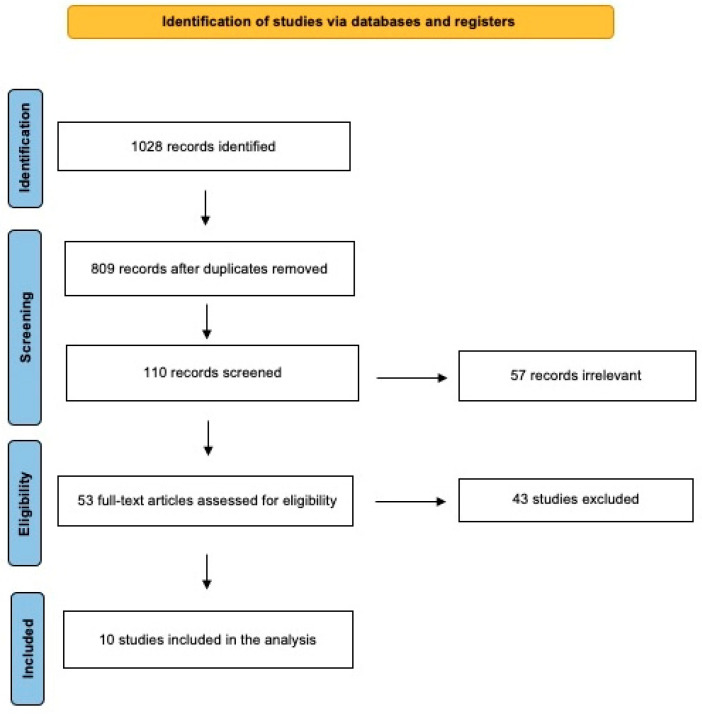
The literature review performed using PRISMA guidelines.

The literature review was performed using the Preferred Reporting Items for Systematic Reviews and Meta-analysis (PRISMA) guidelines for scoping review (Figure 1) [6,7].

The use of the keywords “mitochondrial disease”, “deafness”, “hearing loss” and “single-sided deafness” identified 1028 articles on this topic. After the removal of duplicates and after a critical evaluation of the abstracts, 110 records were selected from 809 papers. Of these, 57 were irrelevant, while 53 were assessed for eligibility. Inclusion criteria were (i) records of diagnosed mitochondrial disease and (ii) hearing loss with at least 1 patient with asymmetric hearing impairment. Patients with other diseases causing sensorineural hearing impairment or mitochondrial disease without hearing involvement were also excluded from the study. Additionally, papers including patients with conductive or mixed hearing loss were excluded from the study.

Data on age, mtDNA mutation, grade of hearing impairment, collateral symptoms, and follow-up were collected and compared. The degree of hearing loss was classified according to its severity into mild (21–40 dB HL), moderate (41–70 dB HL), severe (71–90 dB HL) and profound (>90 dB HL), based on British Society of Audiology criteria [8].

After the full-text review, 10 articles were included in this review, giving a total of 160 patients with mitochondrial disease and hearing loss of which 25 patients presented with asymmetrical hearing impairment [1,2,3,4,5,9,10,11,12,13].

## 3. Results

A total of 160 patients was found to have mitochondrial disease with hearing impairment, and 25 of them had mitochondrial disease with asymmetric hearing loss. The results of the review process are summarized in Table 1. Audiological findings are summarized in Table 2.

The patients’ age ranged from 0 to 58.3 (considering all hearing losses), and the most frequently found genetic mutation was A3243G, which is also the main mutation associated with MELAS syndrome.

The A3243G mutation is also the mutation noted in the highest number of patients with asymmetric presentation, considering the total number of patients with hearing loss [11].

The diagnosis of hearing loss was achieved in all of the studies using pure tone audiometry (PTA), except for the paper by Sakai et al. in which conditioned response audiometry (COR) was performed due to the young age and the developmental delay of the patient under investigation [10]. This type of hearing investigation was the only performed in two cases.

The second most performed examination was auditory brainstem response (ABR), reported in eight cases. Speech audiometry was also performed in six papers, as well as otoacoustic emissions (EOAEs). Tympanometry with stapedial reflexes testing was performed in four cases, while electrocochleography was performed in only one case.

The papers with the most comprehensive hearing assessment included the following (i) Zwirner et al. performed a hearing assessment through PTA, speech audiometry, tympanometry and reflex testing, ABR, EOAEs, and DPOAEs; and (ii) Sue et al. performed PTA, speech audiometry, tympanometry and reflex testing, ABR, electrocochleography and EOAEs [4,11].

Among the patients selected in the study, six had unilateral hearing loss of which three were mild, two moderate, and one profound. Ten patients were reported to be affected by mild hearing loss on one side and by moderate (*n* = 7), severe (*n* = 1), and profound (*n* = 2) on the other side. One patient with marked auditory asymmetry presented hearing loss of a mild grade in one ear and profound in the contralateral ear. In seven patients, moderate hearing loss was present on one side. Of these, five presented severe hearing loss in the contralateral ear, and two cases were profound. Three patients complained of severe hearing loss on one side and profound on the other side.

Unfortunately, the age of onset and the audiometric shape are information not uniformly and relevantly reported by all papers as the purpose of these studies was mainly to focus on other issues than hearing loss.

Associated diseases described within the included patients were mainly ocular and muscular. Ocular manifestations were the symptoms most frequently associated with hearing loss and were found in seven out of 25 patients. Ocular manifestations included retinitis pigmentosa (three patients), optic atrophy (two patient) and ophthalmoplegia (two patients). Due to the small sample size, it is difficult to correlate the ocular abnormalities with a specific genetic alteration.

In the sample collected from the review, three patients had muscle weakness, and one had a mitochondrial myopathy. Furthermore, epileptic episodes were reported in three cases. Of these, one was attributable to MERFF syndrome.

Palmoplantar keratoderma was found in only one case reported in the paper by Zhang et al. [3].

In the cases reported by Joo et al., the A1555G and G7444A mutations were found and were attributed to the asymmetric hearing loss phenotypes (including profound hearing loss on one side and mild hearing loss on the other) and the lack of concomitant other symptoms [1].

Concerning audiological follow-up, the information gathered from this review is scarce. When mentioned, follow-up length is not specifically indicated. Furthermore, it focuses on other symptoms related to the mitochondrial disease and not specifically to hearing loss.

## 4. Discussion

mtDNA mutations have been found in 5–10% of patients with postlingual nonsyndromic hearing loss [2]. The 37 known genes contained in mtDNAs represent 0.5% of the nucleated somatic cell DNA [2]. There are more than 1500 mitochondrial proteins involved in ATP synthesis; mutations in the genes coding for these proteins can lead to mild to severe clinical manifestations [14].

Mitochondrial diseases may determine alterations such as subsarcolemmal accumulation of mitochondria as noted in red ragged fibers; biochemical imbalances, such as impaired ATP production; or genetic abnormalities [2]. Since all tissues retain mitochondria, all are potential targets of mtDNA alterations; however, tissues with increased metabolism are typically more susceptible [13,15]. As mitochondria are responsible for energy production, the first targets of their disruption are high energy-consuming organs and systems, such as the nervous system, retina, cochlea, muscle, kidney, and liver [3,4].

The stria vascularis has a high level of metabolic activity. In fact, to maintain endolymphatic ion homeostasis, numerous Na^+^ and K^+^-ATPase pumps are involved, and they require continuous supply of ATP to function [13]. The outer hair cells, which are responsible for amplifying local sound stimuli, and the inner hair cell are also highly metabolically active [13]. Therefore, hair cells and stria vascularis are the first to be damaged in cases of deficiencies in intracellular ATP production due to mitochondrial dysfunction [13]. Thus, the cochlea is a major target of mitochondrial dysfunction, and sensorineural hearing loss is a symptom that is frequently present in mitochondrial disorders, both in syndromic and isolated forms [13].

Mitochondrial diseases have a heterogeneous presentation and a progressive course [4]. One of the challenges of mitochondrial diseases is the marked clinical and genetic variability, as the disease manifestations can arise at any age and with any symptom; these features frequently delay diagnosis, especially in adults [14]. The broad range of ages evidenced in this review also indicates variability in clinical presentation.

In vitro studies have shown that a heteroplasmic mtDNA defect is expressed only when the percentage exceeds a defined threshold that varies among individuals and depends on the mtDNA defect. This variability explains all the different clinical phenotypes [5].

Mitochondrial disorders can present in syndromic (i.e., with known clinical and/or metabolic patterns) or nonsyndromic forms [2,5]. Several mitochondrial alterations with syndromic features are known to be associated with hearing loss, which may be the onset symptom of the disease [4].

Kearns-Sayre syndrome (KSS) is frequently sporadic and is characterized by early onset progressive ophthalmoplegia and retinopathy. Other symptoms are short stature, cerebellar changes, tubulopathy, hematologic disorders, and hearing loss [4]. KSS is defined using a diagnostic triad: onset before age 20, progressive ophthalmoplegia, and pigmentary retinopathy [16]. The diagnosis is confirmed when, in addition to the triad, at least one of the following conditions is present: complete heart block, cerebellar ataxia, cerebrospinal fluid (CSF) protein greater than 100 mg/dL, dementia, endocrine abnormalities, and hearing loss [16]. No cases of KSS were found in the case series collected from this review, but we still believe that it falls within the syndromic mitochondrial disorders that should be known and considered in the differential diagnosis in the presence of hearing loss since KSS shares the same symptoms as other mitochondrial disorders associated with hearing loss.

MELAS (mitochondrial encephalomyopathy, lactic acidosis, and stroke-like episodes) syndrome is associated with neurological disorders, retinopathy, ophthalmoplegia, and hearing loss [4]. It is a disease with multi-organ manifestations that includes stroke-like episodes, diabetes, dementia, lactic acidemia, recurrent headaches, hearing loss, epilepsy, myopathy, and short stature. The most common genetic abnormality related to MELAS syndrome is the A3243G mutation in the MT-TL1 gene (tRNA leucine), which encodes mitochondrial tRNA [17]. This mutation causes impaired mitochondrial translation and protein synthesis, including subunits of the mitochondrial electron transport chain complex, resulting in altered mitochondrial energy production [17]. Other mutations associated with MELAS syndrome are the mtDNA point mutations A3252G, C3256T, T3271C, T3291C, and A11084G [4]. The inability of dysfunctional mitochondria to produce adequate energy to meet the needs of different tissues and organs results in the multi-organ manifestation described. Mitochondrial energy defects can also enhance the proliferation of mitochondria in the smooth muscle and endothelial cells of small blood vessels, resulting in angiopathy and impaired blood perfusion in the microvasculature of different organs. All these events could explain the complications observed in MELAS syndrome, including stroke episodes [17].

Furthermore, according to this review findings, patients with the A3243G mutation associated with MELAS are most likely to present with a clinical picture of asymmetric hearing loss. When these features are present, this mutation should be investigated. The mutation can also manifest as sensorineural hearing loss with diabetes mellitus as the only symptom in the absence of neurological disorders [18].

MERRF (myoclonus epilepsy with ragged-red fibers) syndrome is a multisystem mitochondrial syndromic disorder characterized by the onset of progressive myoclonus and seizures [19]. MERRF is also known as Fukuhara syndrome, named after the author who first described it in detail in 1980 [18].

MERRF is caused by genetic abnormalities in mtDNA; the most common mutation identified in more than 75% of patients is A8344G in the tRNA gene (Lys). The MERRF-associated A8344G mutation was also found in the patients highlighted in this review. On muscle biopsy, the presence of muscle fibers with pathognomonic red streaks is distinctive [19]. MERRF also has neurological features such as ataxia, dementia, and optic atrophy associated with hearing loss [4].

Mitochondrial hearing loss can also be observed in nonsyndromic manifestations that can be caused by mitochondrial transfer RNA mutations [3]. Specifically, the A1555G mutation in the 12s subunit of ribosomal RNA (which is the most common mitochondrial variant associated with hearing loss), a point mutation A3243G in the tRNA^Leu^ gene, and four different mutations in tRNA^(Ser)(UCN)^ were identified as causative of nonsyndromic mitochondrial hearing loss [1,10]. Other mutations identified in the literature in small groups of patients are C7472T in tRNA^(Ser)(UCN)^, T7512C in tRNA^(Ser)(UCN)^, and T7510C in tRNA^(Ser)(UCN)^ [10].

Concerning the main findings within the selected studies of this review, the 43 patients reported by Zhang et al. with hearing loss due to mitochondrial pathology showed an almost exclusive auditory clinical presentation with some cases of palmoplantar keratoderma [3]. Since it has no associated symptomatology, this mutation should be carefully considered in individuals with hearing loss alone. According to the observations of Zhang et al., the A7445G and T75101C mutations are of variable penetrance, with 15–100% noted for the former and 61–100% for the latter. Both mutations have an early onset, so an early and effective diagnosis may allow a therapeutic strategy to be established [3].

Zwirner et al. reported on 12 children with early onset mitochondrial disease in syndromic patterns, describing two patients with asymmetrical/mild-moderate presentation. In one of these patients, hearing loss was the onset symptom, and, overall, there was an average worsening 10.7 dB per year [4]. This rate of hearing deterioration was also observed in the studies by Chennupati et al. [2]. Both cochlear and retrocochlear hearing loss were observed, and the authors suggested that the cochlea is the most sensitive, becoming damaged earlier that other tissues [4].

Leruez et al. studied 21 patients with OPA1 mutations, reporting on one patient with asymmetric hearing loss (mild on one side and severe on the other) with a low age of onset. The onset occurred at 4 years of age, which was earlier than that noted for visual impairment and also associated with the mitochondrial disorder. In case of OPA1 mutations, it was observed that optic atrophy and unilateral hearing loss presentations were present in 62% of cases. In addition, hearing loss occurred before visual involvement in 54% of cases, thus an early diagnosis is crucial [9].

Ocular manifestations appear most likely to be associated with hearing loss in mitochondrial diseases. Therefore, when ocular manifestations and hearing loss (even if asymmetrical) present together, a mitochondrial disorder should be considered among the differential diagnosis.

The case series by Chinnery et al. reported that of 23 patients with hearing loss and mitochondrial disease, four had a definite hearing asymmetry. Three of them carried the A3243G mutation associated with MELAS syndrome [5]. This study also highlighted the variability in hearing loss presentation in MELAS syndrome, as also confirmed by Chennupati et al. [2].

According to Sue et al., who studied 18 patients with MELAS syndrome (and 6 of these had asymmetric hearing loss), hearing impairment is often the result of cochlear damage; therefore, these patients could effectively benefit from cochlear implantation [11]. For this reason, early diagnosis in the pediatric population could provide the best hearing outcome before this deficit compromises the psychomotor development [20].

Sakai et al. studied two sisters with a history of seizures at birth and the subsequent development of retinitis pigmentosa and muscle weakness [10]. The older sister’s medical records allowed an early diagnosis in the younger sister as her hearing assessment, from the age of eight months to three years, showed a progression from hearing loss to deafness [10]. The early diagnosis, which was based on the detection of the T8993G mutation in the mother and in the older sister, allowed the prediction of the progressive deterioration of her hearing.

Joo et al. identified the G7444A mutation as a cause of sudden-onset asymmetrical hearing loss. The authors pointed out that the mutation itself is poorly capable of causing a pathological phenotype of hearing loss, and it requires the additional presence of the A1555G mutation, aminoglycoside exposure, or other factors related to ethnic and genetic background [1].

Therefore, it is essential to recognize isolated symptoms or constellations of symptoms suggestive of mitochondrial pathology, therefore requiring specific genetic testing. Based on the data of this review, the symptoms of primary concern are neurological signs, ocular signs, muscle weakness, epilepsy, diabetes, and palmoplantar keratoderma. In addition, as suggested by Koleilat et al., screening for sensorineural hearing loss could facilitate the early detection of mitochondrial disease [21].

Another useful finding is related to the length of audiological follow-up in patients with mitochondrial disease. In fact, data on this feature are lacking, and the evolution of hearing loss, especially in the pediatric population, should be investigated carefully to assess further eventual threshold deteriorations.

This study has several limitations. First, there are a small number of articles in the literature regarding mitochondrial disease, as it is a rare condition. Moreover, even rarer is the asymmetrical presentation of this type of hearing loss; this fact further decreases the number of articles in the literature available on the topic. The low number of articles also corresponds to the low number of patients and limited data from different studies. Furthermore, most studies do not focus on hearing loss because mitochondrial disorders often are multi-organ diseases with a complex clinical picture. Therefore, focusing on hearing loss is not critical. This often leads to an inaccurate assessment of auditory function, and limited information is available on the follow-up and progression of hearing loss in mitochondrial diseases in the current literature.

To the best of our knowledge, this is the first review study to analyze syndromic and nonsyndromic mitochondrial diseases presenting with asymmetric hearing loss. This work emphasizes that these clinical entities can occur, as per the available literature.

## 5. Conclusions

Mitochondrial disorders associated with hearing loss are rare and may belong to syndromic or nonsyndromic diseases. Hearing loss, in the case of mitochondrial disorders, normally has a symmetrical presentation and progressive course; however, there are documented cases of asymmetrical presentation that can hamper the formulation of a correct diagnosis. Thus, when assessing unilateral or asymmetrical hearing loss, it is also important to consider a mitochondrial disorder in the differential diagnosis. The association with the syndromic picture may be highly suggestive of mitochondrial disease, but it is also possible to encounter this entity as an isolated hearing loss event or with a limited number of associated symptoms.

Further follow-up studies are necessary to better comprehend the evolution of hearing loss associated with mitochondrial disease and to predict the optimal therapeutic timing. In our opinion, since this is a spectrum of rare diseases, collaboration among multiple centers and specialists is critical to obtain as much data as possible.

## Figures and Tables

**Table 1:** Identified papers with mitochondrial disease and hearing loss with unilateral presentation: audiological, genetic, and clinical features.

Author	Number of Patients with Asymmetric Hearing Loss (Patients Cited in the Study)	Average Age (Range)	Genetic Mutation	Other Symptoms
Chennupati et al. [2]	1/15	5.5 (0–17)	1 pt A8344G MERRF	MERFF
Chinnery et al. [5]	4/23	45.6 (25–76)	3 pts A3243G 1 pt T10010C + A5656G	MELAS
Forli et al. [13]	3/8	58.3 (54–64)	Multiple mt DNA deletions	2 pts ophthalmoplegia 1 pt mitochondrial myopathy
Joo et al. [1]	2/12	39.8	1 pt A1555G 1 pt G7444A	None
Leruez et al. [9]	1/21	38.7 (12–73)	1 pt OPA1	Optic atrophy
Roubertie et al. [12]	2/6	40	1 pt G1334A 1 pt C2708 del TTAG	Optic atrophy
Sakai et al. [10]	1/2	7	1 pt T8993G	Muscle weakness, retinitis pigmentosa, epileptic seizures
Sue et al. [11]	6/18	40 (12–72)	6 pts A3243G MELAS	All pts epilepsy, ataxia, diabetes, muscle weakness, migraine, short stature
Zhang et al. [3]	3/43	37.6 (10–53)	2 pts A7445G 1 pt T7510C	1 pt palmoplantar keratoderma
Zwirner et al. [4]	2/12	10.8 (3–20)	Unspecified deletion of mtDNA	1 pt ptosis, pigmentary retinopathy, short stature, cerebellar symptoms, mental retardation, muscular weakness 1 pt ptosis, pigmentary retinopathy

**Table 2:** Audiological features of the patients with asymmetrical hearing loss recorded in the review. Abbreviations: pt (patient).

Author	Left Ear: Grade of Hearing Loss	Right Ear: Grade of Hearing Loss
Chennupati et al. [2]	Moderate	Mild
Chinnery et al. [5]	Moderate	Normal hearing
	Moderate	Mild
	Moderate	Severe
	Profound	Moderate
Forli et al. [13]	Profound	Normal hearing
	Normal hearing	Mild
	Severe	Moderate
Joo et al. [1]	Mild	Normal hearing
	Profound (sudden)	Mild
Leruez et al. [9]	Mild	Severe
Roubertie et al. [12]	Severe	Moderate
	Moderate	Mild
Sakai et al. [10]	Severe	Profound
Sue et al. [11]	Moderate	Severe
	Normal hearing	Mild
	Mild	Moderate
	Mild	Moderate
	Mild	Profound
	Normal hearing	Moderate
Zhang et al. [3]	Severe	Profound
	Severe	Profound
	Moderate	Severe
Zwirner et al. [4]	Mild	Moderate
	Mild	Moderate
